# No difference in biomechanical properties of simple, horizontal mattress, and double row repair in Bankart repair: a systematic review and meta-analysis of biomechanical studies

**DOI:** 10.1186/s12891-023-06864-2

**Published:** 2023-09-28

**Authors:** Jun-Ho Kim, Bon-Ki Koo, Ki Hyeok Ku, Myung Seo Kim

**Affiliations:** Department of Orthopedic Surgery, Kyung Hee University Hospital at Gangdong, School of Medicine, Kyung Hee University, 892 Dongnam-ro, Gangdong-gu, Seoul, 05278 Korea

**Keywords:** Bankart repair, Simple, Horizontal mattress, Double row, Biomechanical results

## Abstract

**Background:**

Arthroscopic Bankart repair is the most common procedure in patients with anterior shoulder instability. Various repair techniques using suture anchors have been used to improve the strength of fixation and surgical outcomes in arthroscopic Bankart surgery. However, evidence regarding which method is superior is lacking. This systematic review and meta-analysis study was designed to compare the biomechanical results of simple versus horizontal mattress versus double-row mattress for Bankart repair.

**Methods:**

A systematic search of the MEDLINE, Embase, and Cochrane Library databases was performed to identify comparative biomechanical studies comparing the simple, horizontal mattress, and double-row techniques commonly used in Bankart repair for anterior shoulder instability. Biomechanical results included the ultimate load to failure, stiffness, cyclic displacement, and mode of failure after the ultimate load. The methodological quality was assessed based on the Quality Appraisal for Cadaveric Studies (QUACS) scale for biomechanical studies.

**Results:**

Six biomechanical studies comprising 125 human cadavers were included in this systematic review. In biomechanical studies comparing simple and horizontal mattress repair and biomechanical studies comparing simple and double-row repair, there were no significant differences in the ultimate load to failure, stiffness, or cyclic displacement between the repair methods. The median QUACS scale was 11.5 with a range from 10 to 12, indicating a low risk of bias.

**Conclusion:**

There was no biomechanically significant difference between the simple, horizontal mattress, and double-row methods in Bankart repair. Clinical evidence such as prospective randomized controlled trials should be conducted to evaluate clinical outcomes according to the various repair methods.

**Level of evidence:**

Systematic review, Therapeutic level IV.

**Supplementary Information:**

The online version contains supplementary material available at 10.1186/s12891-023-06864-2.

## Background

 Both conservative and surgical treatments may be applied in patients with anterior shoulder instability [1–3]. During Bankart repair surgery, it is important to restore the labrum, which is one of the key structures contributing to shoulder instability, to the rim of the glenoid [4, 5]. In the past, open procedures using bone tunnels have been performed [6], but arthroscopic Bankart repair surgery is currently performed to obtain improved visualization of labrum and adjacent structure [7–9].

In arthroscopic Bankart repair, a suture anchor is used to form a secure knot that attaches the labrum to the rim of the glenoid [4]. Although arthroscopic Bankart repair is the most common procedure, with few complications and good functional outcomes, the postoperative recurrence rate can still reach approximately 20% [8, 10]. As chronic instability and early onset osteoarthritis may arise when labral fixation fails and re-dislocation occurs after surgery [11], various repair techniques have been developed and performed to improve the strength of fixation and surgical outcomes [5, 12, 13].

Cadaveric studies comparing simple and mattress repair techniques which are mainly used in Bankart repair have reported that the biomechanical strength of the two methods did not show significant difference [7, 14, 15]. In addition, studies comparing simple single-row and double-row repair techniques have shown different results [5, 7]. Judson et al. reported that there was no difference in the load to failure, cyclic displacement, and cyclic stiffness between the two techniques [7]. However, McDonald et al. reported that double-row repair resulted in a more secure fixation than simple single-row repair [5]. Therefore, a clear consensus is yet to be reached.

Biomechanical evidence for the repair technique is needed to allow clinicians to fix the labrum more firmly during Bankart repair surgery, but, currently, no systematic review data is available. The purpose of this study was to conduct a meta-analysis of the current literature comparing the simple, horizontal mattress, and double-row techniques commonly used in Bankart repair for anterior shoulder instability. The hypothesis was that the simple repair method would not show lower biomechanical strength than the horizontal mattress or double-row methods in Bankart repair.

## Methods

### Literature search

This systematic review and meta-analysis was performed in accordance with the Preferred Reporting Items for Systematic Reviews and Meta-analyses (PRISMA) guidelines and algorithms [16]. The protocol for review was registered in the International Prospective Register of Systematic Reviews (PROSPERO, registration no. CRD).

Two independent reviewers (J-H. K and M-S.K) systematically searched for articles in the PubMed (MEDLINE), EMBASE, and Cochrane Library from study inception to March 1, 2022, using an *a priori* search strategy. The following keywords were used in the search: “shoulder,” “capsulolabral,” “Bankart,” “horizontal mattress,” “simple,” “double row,” “single row,” and “repair” aided using Boolean operators “AND” or “OR.” The bibliographies of the initially retrieved studies were manually cross-checked to identify additional relevant articles that could have been missed by electronic searches. No language restrictions were applied.

### Study selection

Two reviewers (J-H. K and M-S.K) independently screened the titles and abstracts of the retrieved articles; full manuscripts were reviewed if the abstract provided insufficient data for inclusion in the study. Disagreements were resolved through discussion. Studies were included in the current analysis if they met the patients, intervention, comparison, outcome, study design (PICOS) criteria (Table 1) [17].

**Table 1:** Inclusion and Exclusion criteria based on PICO^a^

PICO	Inclusion criteria	Exclusion criteria
Population	Human cadaver with Bankart lesion	Animal study
Intervention	Horizontal mattress suture repair or double-row suture repair	Repair with remplissage
Comparison	Simple suture repair or single-row suture repair	
Outcome	Biomechanical outcomes	Anatomical outcomes such as contact pressure or area

^a^*PICO* population intervention comparison outcome

The exclusion criteria were: (1) conference or (2) clinical trial abstracts, (3) insufficient statistics or inability to reproduce statistics, (4) animal studies (5) concomitant procedures such as remplissage, (6) anatomical outcomes such as contact pressure or area. As only one clinical study was found according to a pilot systematic search, [18] only biomechanical studies were included for the current systematic review.

### Assessment of Methodological Quality

The methodological quality was assessed by two reviewers (J-H. K and M-S.K) based on the Quality Appraisal for Cadaveric Studies (QUACS) scale for biomechanical studies, which consists of 13 items with a maximum score of 13 [19]. The QUACS scale is reliable and has a strong construct validity for biomechanical research [19]. Publication bias was not assessed as it is generally not considered necessary if fewer than ten studies are being compared [20].

### Data extraction

The same reviewers independently collected available data from the included studies, and any disagreement was resolved by discussion. The basic characteristics of the study (author, journal, year of publication, sample size, and LOE), details of patient characteristics (mean age, sex proportion, and mean bone mineral density), and details of the surgical technique for Bankart repair were collected. For outcome measurements in biomechanical studies, any of the variables reported in more than two studies were collected, such as the ultimate load to failure, stiffness, cyclic displacement, and mode of failure after the ultimate load. For papers with missing data, we attempted to contact the author of the article first; if this failed, we calculated the missing values from other available data using formulas in the Cochrane Handbook for Systematic Reviews of Interventions [20].

### Statistical analysis

The primary outcome of the systematic review was to evaluate Bankart repair using various techniques. If possible, a meta-analysis was performed to show the standardized mean difference (SMD) with 95% confidence interval (CI) for continuous variables and the odds ratio (OR) with 95% CI for dichotomous variables. If a meta-analysis was not possible due to a lack of variables, a qualitative description of the outcome was performed. Heterogeneity was assessed by estimating the proportion of between-study inconsistencies due to actual differences between studies using the *I*^2^ statistic [20]. A random-effects meta-analysis was performed to pool the outcomes across the included studies. Forest plots were used to show outcomes, the pooled estimate of effect, and the overall summary effect of each study, and were constructed using RevMan version 5.4 (Copenhagen, The Cochrane Collaboration). Statistical significance was set at *P* < .05.

## Results

### Identification of studies

**Fig. 1:**
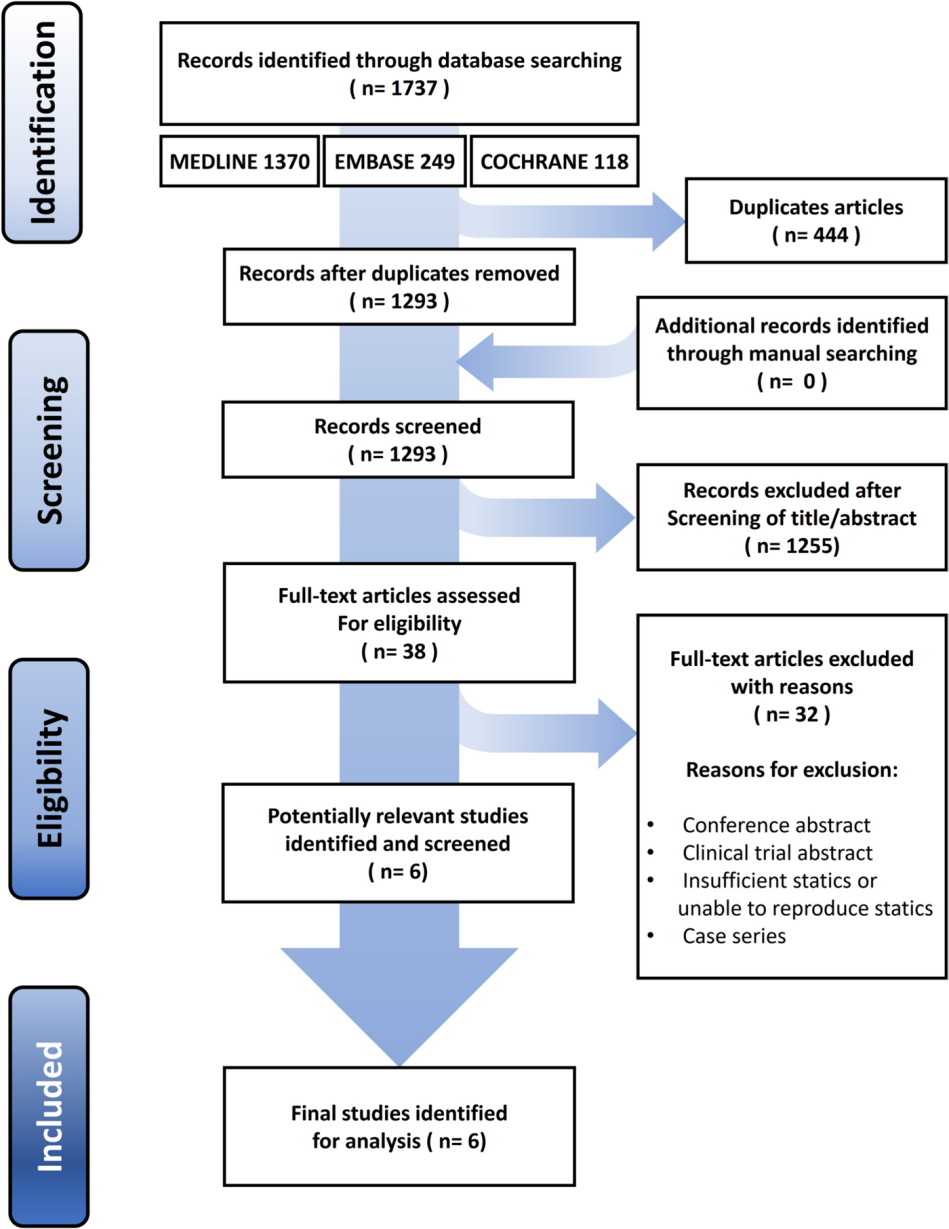
PRISMA (Preferred Reporting Items for Systematic reviews and Meta-analyses) flow diagram showing the identification and selection of studies

The initial electronic search yielded 1737 studies. After removing 444 duplicates, 1293 studies remained. Of these, 1255 were excluded after reading the title or abstract and 31 were excluded after a full-text review. Ultimately, six biomechanical studies [5, 7, 14, 15, 21, 22] were included in this systematic review (Fig. 1).

### Assessment of methodological quality

The QUACS scale was used to assess biomechanical studies, with a maximum score of 13 points. The median QUACS scale was 11.5 with a range from 10 to 12, indicating a low risk of bias (Table 2).

### Study characteristics

All six biomechanical studies were controlled laboratory studies. Of these six studies, three [14, 15, 21] compared horizontal mattress repair with simple repair, two [5, 22] compared double-row repair with simple single-row repair, and one [7] compared horizontal mattress repair, simple single-row repair, and double-row repair. The median QUACS scale for assessment of methodological quality was 11.5 with a range from 10 to 12, indicating a low risk of bias (Table 2 and Supplementary table). A total of 125 human cadavers with Bankart lesions were included in the final analysis. Detailed characteristics of the included biomechanical studies are presented in Table 2. All six biomechanical studies used 3.0 mm bioabsorbable suture anchors (Arthrex) and the No. 2 FiberWire suture material (Arthrex) for Bankart repair. Further, two studies [15, 22] used two anchors and four [5, 7, 14, 21] used three anchors. The detailed anchor characteristics used in the biomechanical studies are presented in Table 3.

**Table 2:** General characteristics of the included biomechanical studies^a^

Study	Journal	Year	Study Design	Technique: Sample Size	Age, year	Sex, M:F	BMD, g/cm^2^	QUACS scale
Nho et al.	AJSM	2010	CLS	HM: 5 Simple: 5	59.0 ± 10.8 57.8 ± 13.6	4:1 2:3	0.67 ± 0.1 0.61 ± 0.1	11
Spiegl et al.	AJSM	2014	CLS	DR: 14 SR: 14	54.3 (44–64)	9:5	0.49 (0.46–0.61)	11
McDonald et al.	Arthroscopy	2016	CLS	DR: 6 SR: 6	61 (19–73)	NR	NR	10
Judson et al.	AJSM	2017	CLS	HM: 6 DR: 6 Simple (SR): 6	63.3 ± 8.9	NR	No difference among groups.	12
Lacheta et al.	Arthroscopy	2020	CLS	HM: 6 Simple: 6	56.4 (37–60)	NR	NR	12
Miskovsky et al.	OJSM	2020	CLS	HM: 25 Simple: 20	(30–50)	NR	NR	12

*BMD* Bone mineral density, *QUACS* Quality Appraisal for Cadaveric Studies), *AJSM* American Journal of Sports Medicine, *OJSM* Orthopaedic Journal of Sports Medicine, *CLS* Controlled laboratory study, *HM* Horizontal mattress, *DR* double-row, *SR* single-row, *NR* not reported

^a^Values present as mean ± standard deviation (range) and number

**Table 3:** Anchor characteristics of the included biomechanical studies^a^

Study	Anchor	No. of Anchor	Position of Anchor	Suture Material
Nho et al.	3.0 mm bioabsorbable suture anchor (PEEK Suture Tak, Arthrex)	2	4 and 5 o’ clock	No. 2 FiberWire (Arthrex)
Spiegl et al.	3.0 mm bioabsorbable suture anchor (BioComposite SutureTak, Arthrex)	2	1:30 and 4:30 clock	No. 2 FiberWire (Arthrex)
McDonald et al.	3.0 mm bioabsorbable suture anchor (BioComposite SutureTak, Arthrex) / 2.9 mm Pushlock (Arthrex) for DR technique	3	3, 4, and 5 o’clock	No. 2 FiberWire (Arthrex)
Judson et al.	3.0 mm bioabsorbable suture anchor (Bio-SutureTak, Arthrex) / 2.9 mm Pushlock (Arthrex) for DR technique	3	2:30, 4, and 5 o’clock	No. 2 FiberWire (Arthrex)
Lacheta et al.	3.0 mm bioabsorbable suture anchor (Bio-SutureTak, Arthrex)	3	3:30, 4:30, and 5:30	No. 2 FiberWire (Arthrex)
Miskovsky et al.	3.0 mm bioabsorbable suture anchor (Bio-SutureTak, Arthrex)	3	3, 4:30, and 6 o’clock	No. 2 FiberWire (Arthrex)

^a^Values present as mean ± standard deviation (range) and number

### Horizontal mattress repair versus simple repair

**Fig. 2:**
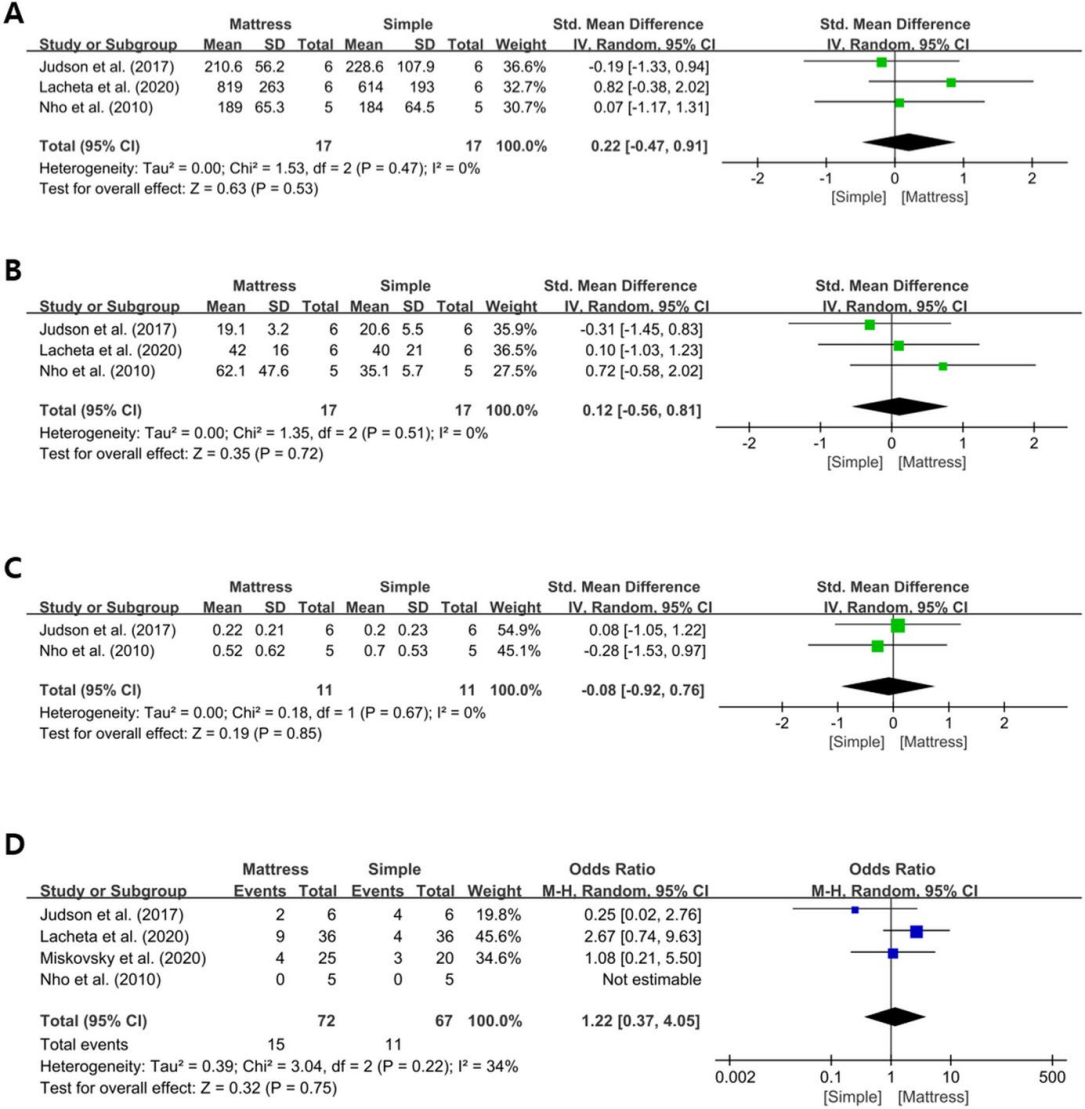
**A**-**D** Forest plot showing the standard mean differences in biomechanical outcomes comparing horizontal mattress suture with simple suture techniques. **A** Ultimate load to failure. **B** Stiffness. **C** Cyclic displacement. **D** Mode of failure. CI, confidence interval; IV, inverse variance; SD, standard deviation

Four biomechanical studies [7, 14, 15, 21] compared the stability after Bankart repair between horizontal mattress and simple stitch configurations. Of these four studies, three [7, 14, 15] reported the ultimate load to failure, and there was no significant difference between the two techniques (SMD, 0.22; 95% CI, -0.47 to 0.91; *I*^2^, 0%; Z = 0.63; *P* = .53) (Fig. 2A). Three studies [7, 14, 15] also reported stiffness, and no significant difference was found between the two techniques (SMD, 0.12; 95% CI, -0.56 to 0.81; *I*^2^, 0%; Z = 0.35; *P* = .72) (Fig. 2B). Of the four studies, two [7, 15] reported cyclic displacement, and there was no difference between the two techniques (SMD, -0.08; 95% CI, -0.92 to 0.76; *I*^2^, 0%; Z = 0.19; *P* = .85) (Fig. 2C). All four studies [7, 14, 15, 21] reported the failure mode after the ultimate load. The pooled incidence of suture or knot failure showed no significant difference between the two techniques (OR, 1.22; 95% CI, 0.37 to 4.05; *I*^2^, 34%; Z = 0.32; *P* = .75) (Fig. 2D).

### Double-row repair versus simple single-row repair

**Fig. 3:**
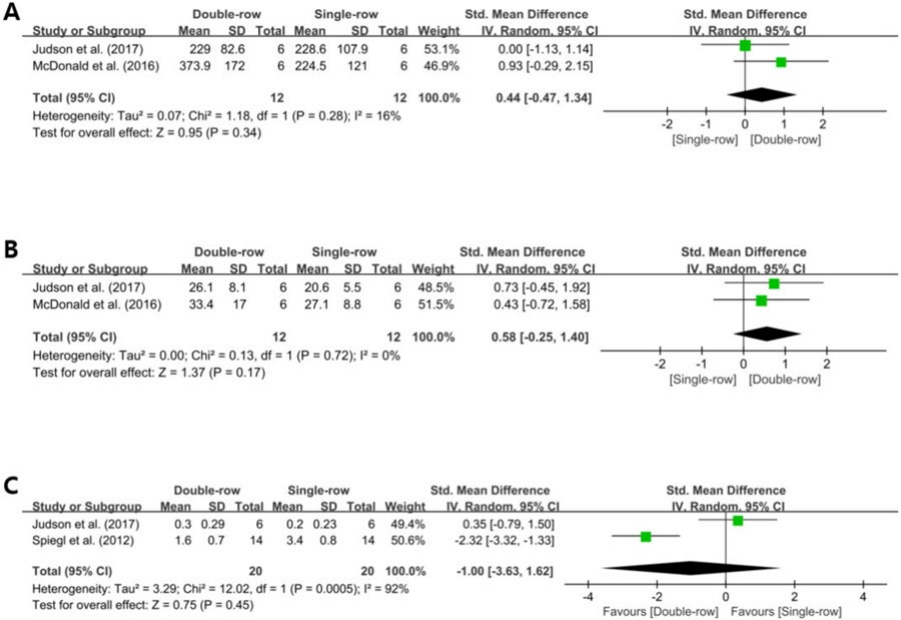
** A-C** Forest plot showing the standard mean differences in biomechanical outcomes comparing double-row repair with simple single-row repair techniques. **A** Ultimate load to failure. **B** Stiffness. **C** Cyclic displacement. CI, confidence interval; IV, inverse variance; SD, standard deviation

Three biomechanical studies [5, 7, 22] compared the stability after Bankart repair between the double-row and simple single-row repair techniques. Of these, two studies [5, 7] reported the ultimate load to failure, showing no significant difference between the two techniques (SMD, 0.44; 95% CI, -0.47 to 1.34; *I*^2^, 16%; Z = 0.95; *P* = .34) (Fig. 3A). Two studies [5, 7] further reported stiffness, with no significant difference found between the two techniques (SMD, 0.58; 95% CI, -0.25 to 1.40; *I*^2^, 0%; Z = 1.37; *P* = .17) (Fig. 3B). For cyclic displacement, two studies reported no significant difference between the two techniques (SMD, -1.0; 95% CI, -3.63 to 1.62; *I*^2^, 92%; Z = 0.75; *P* = .45) (Fig. 3C).

## Discussion

In this study, the simple repair method did not show lower biomechanical strength than the horizontal mattress or double-row methods during Bankart repair. Furthermore, the ultimate load to failure, stability, and cyclic disposition showed no significant differences between the three repair techniques.

In patients with anterior shoulder instability, various surgical methods, such as the trans-glenoid suture technique and bioabsorbable tack, have been performed in the past, but a high recurrence rate has been reported even after surgery [23, 24]. With the introduction and wide implementation of arthroscopic Bankart repair using suture anchors, several studies have reported lower recurrence rates and better clinical outcomes than before [25–28]. However, variable recurrence rates of up to 20% have been reported [29]. If instability occurs again after surgery, bone defects in the glenoid and humerus could increase due to recurrent dislocation, and osteoarthritis may occur [11, 30]. Indeed, Buck et al. reported a possibility of atrophy of the rotator cuff muscle if revision Bankart repair is performed because of recurrent instability [31]. Therefore, various suture anchors and repair techniques should be used to firmly fix the torn labrum to the glenoid during surgical treatment [12, 14, 32].

Several studies have reported the use of various suture anchors during Bankart repair [33–35]. Lee et al. compared patients who used all-suture anchors during Bankart repair with those who used biodegradable suture anchors and reported no significant difference between the clinical outcomes and recurrence rates [35]. Furthermore, Jin et al. reported that there was no difference in recurrence instability in patients who underwent Bankart repair using a biocomposite anchor and an all-suture anchor, and both patients showed satisfactory outcomes [34]. According to a systematic review that analyzed the clinical differences depending on the anchor material and type, there was no significant difference in the occurrence of recurrent instability after Bankart repair [36]. However, as no systematic review or meta-analysis has yet compared suture repair techniques in Bankart repair, there is a lack of evidence for clinical reference.

Common methods used during Bankart repair include the simple, horizontal mattress and the double-row technique. In this review, four studies comparing the biomechanical properties of the simple and horizontal mattress methods were analyzed [7, 14, 15, 21]. The pooled mean incidence rate of failure was 16.4% for the simple method and 20.8% for the horizontal mattress method, which was not statistically significant [7, 14, 15, 21]. In most of the included studies (3 of 4 studies), the pooled mean ultimate load to failure (simple, 342.2 N vs. The horizontal mattress, 406.2 N), and stiffness (simple, 31.9 N/mm vs. horizontal mattress, 41.1 N/mm) also showed no difference [7, 14, 15]. In the case of cyclic disposition, it was reported that there was no difference between the two methods in two studies [7, 15]. Three biomechanical studies analyzing simple single-row and double-row repair were further included in analysis [5, 7, 22]. In 2 of 3 studies, the pooled mean ultimate load to failure (simple single-row, 226.6 N vs. double-row, 301.5 N) [5, 7], stiffness (simple single-row, 23.9 N/mm vs. double-row, 29.8 N/mm) [5, 7] and cyclic displacement (simple single-row, 1.8 mm vs. double-row 1.0 mm) [7, 22] showed no significant difference. Based on this finding, this review suggests that the simple, horizontal mattress and double-row methods during Bankart repair show similar biomechanical properties. On the other hand, Spiegl et al. reported that double row repair had smaller cyclic displacement than single row repair, which is thought to be because their Cadaveric study was conducted on bony Bankart lesions, unlike the other two studies. Another Cadaveric study also reported that double row repair was more stable than single row repair in bony Bankart lesions [37]. However, since the outcome measurement (ultimate load to failure, stiffness, cyclic displacement and mode of failure) set in this meta-analysis was not analyzed in their study we did not include it.

This study has several limitations. First, we could find only one study comparing the biomechanical properties of the three repair methods (simple, horizontal mattress, and double-row). In addition, the number of studies analyzed in this systematic review was small. For example, four studies compared the biomechanical properties of simple repair and horizontal mattress repair, and only three studies compared simple single-row and double-row repair. Second, there was no clinical study that directly compared simple, horizontal mattress, and double-row repair in a clinical setting, and even the one clinical study comparing hybrid methods with a simple technique was a retrospective cohort design, and was not a randomized controlled study. In this regard, systematic review was not possible for clinical evidence.

## Conclusion

Based on the systematic review and meta-analysis, the simple, horizontal mattress, and double-row methods in Bankart repair were biomechanically similar in terms of the ultimate load to failure, stiffness, and cyclic displacement. However, clinical evidence such as prospective randomized controlled trials should be conducted to evaluate clinical outcomes according to the various repair methods.

### Supplementary Information

**Additional file 1: Supplementary table 1. **Detailed item and scoring of the QUAC scale.

## Data Availability

The datasets used and/or analyzed during the current study are available from the corresponding author on reasonable request.

## Abbreviations

LOD: Level of evidence
SMD: Standardized mean difference
CI: Confidence interval
OR: Odds ratio

## References

[1] Alkhatib N, Abdullah ASA, AlNouri M, Ahmad Alzobi OZ, Alkaramany E, Ishibashi Y (2022). Short- and long-term outcomes in Bankart repair vs. conservative treatment for first-time anterior shoulder dislocation: a systematic review and meta-analysis of randomized controlled trials. J Shoulder Elbow Surg.

[2] Wang SI (2018). Management of the first-time traumatic anterior shoulder dislocation. Clin Shoulder Elb.

[3] Rhee YG, Cho NS, Cho SH (2009). Traumatic anterior dislocation of the shoulder: factors affecting the progress of the traumatic anterior dislocation. Clin Orthop Surg.

[4] Hagstrom LS, Marzo JM (2013). Simple versus horizontal suture anchor repair of Bankart lesions: which better restores labral anatomy?. Arthroscopy.

[5] McDonald LS, Thompson M, Altchek DW, McGarry MH, Lee TQ, Rocchi VJ (2016). Double-row capsulolabral repair increases load to failure and decreases excessive motion. Arthroscopy.

[6] Rowe CR, Patel D, Southmayd WW (1978). The Bankart procedure: a long-term end-result study. J Bone Joint Surg Am.

[7] Judson CH, Voss A, Obopilwe E, Dyrna F, Arciero RA, Shea KP (2017). An anatomic and biomechanical comparison of Bankart repair configurations. Am J Sports Med.

[8] Hurley ET, Manjunath AK, Bloom DA, Pauzenberger L, Mullett H, Alaia MJ (2020). Arthroscopic Bankart repair versus conservative management for first-time traumatic anterior shoulder instability: a systematic review and meta-analysis. Arthroscopy.

[9] Rashid MS, Arner JW, Millett PJ, Sugaya H, Emery R (2020). The Bankart repair: past, present, and future. J Shoulder Elbow Surg.

[10] Voos JE, Livermore RW, Feeley BT, Altchek DW, Williams RJ, Warren RF (2010). Prospective evaluation of arthroscopic Bankart repairs for anterior instability. Am J Sports Med.

[11] Hovelius L, Saeboe M (2009). Neer award 2008: arthropathy after primary anterior shoulder dislocation–223 shoulders prospectively followed up for twenty-five years. J Shoulder Elbow Surg.

[12] Siripipattanamongkol P, Wongtriratanachai P, Nimkingratana P, Phornphutkul C (2020). Arthroscopic Bankart repair: a matched cohort comparison of the modified Mason Allen method and the simple stitch method. Asia Pac J Sports Med Arthrosc Rehabil Technol.

[13] Yousif MJ, Bicos J (2017). Biomechanical comparison of single- versus double-row capsulolabral repair for shoulder instability: a review. Orthop J Sports Med.

[14] Lacheta L, Brady A, Rosenberg SI, Dornan GJ, Dekker TJ, Anderson N (2020). Biomechanical evaluation of knotless and knotted all-suture anchor repair constructs in 4 Bankart repair configurations. Arthroscopy.

[15] Nho SJ, Frank RM, Van Thiel GS, Wang FC, Wang VM, Provencher MT (2010). A biomechanical analysis of anterior Bankart repair using suture anchors. Am J Sports Med.

[16] Moher D, Shamseer L, Clarke M, Ghersi D, Liberati A, Petticrew M (2015). Preferred reporting items for systematic review and meta-analysis protocols (PRISMA-P) 2015 statement. Syst Rev..

[17] Liberati A, Altman DG, Tetzlaff J, Mulrow C, Gotzsche PC, Ioannidis JP (2009). The PRISMA statement for reporting systematic reviews and meta-analyses of studies that evaluate health care interventions: explanation and elaboration. J Clin Epidemiol.

[18] Lai MC, Ang FHB, Lee KH, Chang CCP, Lie TTD (2019). Hybrid suture technique vs simple suture technique for antero-inferior labral tears: two years’ clinical outcomes. Asia Pac J Sports Med Arthrosc Rehabil Technol.

[19] Wilke J, Krause F, Niederer D, Engeroff T, Nurnberger F, Vogt L (2015). Appraising the methodological quality of cadaveric studies: validation of the QUACS scale. J Anat.

[20] Melsen WG, Bootsma MC, Rovers MM, Bonten MJ (2014). The effects of clinical and statistical heterogeneity on the predictive values of results from meta-analyses. Clin Microbiol Infect.

[21] Miskovsky SN, Sasala LM, Talbot CN, Knapik DM (2020). Differences in failure mode between simple and mattress suture configuration in arthroscopic Bankart repairs: a cadaveric study. Orthop J Sports Med.

[22] Spiegl UJ, Smith SD, Todd JN, Coatney GA, Wijdicks CA, Millett PJ (2014). Biomechanical comparison of arthroscopic single- and double-row repair techniques for acute bony Bankart lesions. Am J Sports Med.

[23] Morgan CD, Bodenstab AB (1987). Arthroscopic Bankart suture repair: technique and early results. Arthroscopy.

[24] Segmuller HE, Hayes MG, Saies AD (1997). Arthroscopic repair of glenolabral injuries with an absorbable fixation device. J Shoulder Elbow Surg.

[25] Marquardt B, Witt KA, Liem D, Steinbeck J, Potzl W (2006). Arthroscopic Bankart repair in traumatic anterior shoulder instability using a suture anchor technique. Arthroscopy.

[26] Kim SH, Ha KI, Kim SH (2002). Bankart repair in traumatic anterior shoulder instability: open versus arthroscopic technique. Arthroscopy.

[27] Vermeulen AE, Landman EBM, Veen EJD, Nienhuis S, Koorevaar CT (2019). Long-term clinical outcome of arthroscopic Bankart repair with suture anchors. J Shoulder Elbow Surg.

[28] Cho HL, Lee CK, Hwang TH, Suh KT, Park JW (2010). Arthroscopic repair of combined Bankart and SLAP lesions: operative techniques and clinical results. Clin Orthop Surg.

[29] Kennedy MI, Murphy C, Dornan GJ, Moatshe G, Chahla J, LaPrade RF (2019). Variability of reporting recurrence after arthroscopic Bankart repair: a call for a standardized study design. Orthop J Sports Med.

[30] Provencher MT (2018). Editorial commentary: is it time to take a stand? When arthroscopic Bankart repair is no longer a viable option for anterior shoulder instability. Arthroscopy.

[31] Buckup J, Welsch F, Hoffmann R, Roessler PP, Schuttler KF, Stein T (2018). Rotator cuff muscular integrity after arthroscopic revision of a Bankart repair. Arch Orthop Trauma Surg.

[32] Connaughton AJ, Kluczynski MA, Marzo JM (2021). Simple versus horizontal mattress suture configuration in Bankart repair. J Orthop.

[33] Ng DZ, Kumar VP (2014). Arthroscopic Bankart repair using knot-tying versus knotless suture anchors: is there a difference?. Arthroscopy.

[34] Jin S, Chun YM (2020). Peri-anchor cyst formation after arthroscopic Bankart repair: comparison between biocomposite suture anchor and all-suture anchor. Clin Shoulder Elb.

[35] Lee JH, Park I, Hyun HS, Kim SW, Shin SJ (2019). Comparison of clinical outcomes and computed tomography analysis for tunnel diameter after arthroscopic Bankart repair with the all-suture anchor and the biodegradable suture anchor. Arthroscopy.

[36] Brown L, Rothermel S, Joshi R, Dhawan A (2017). Recurrent instability after arthroscopic Bankart reconstruction: a systematic review of surgical technical factors. Arthroscopy.

[37] Greenstein AS, Chen RE, Knapp E, Brown AM, Roberts A, Awad HA, Voloshin I (2021). A biomechanical, cadaveric evaluation of single- versus double-row repair techniques on stability of bony Bankart lesions. Am J Sports Med.

