# Supplementary material for: No difference in biomechanical properties of simple, horizontal mattress, and double row repair in Bankart repair: a systematic review and meta-analysis of biomechanical studies
Source: BMC Musculoskelet Disord. 2023 Sep 28;24:765. doi: 10.1186/s12891-023-06864-2 (PMC10536762; doi:10.1186/s12891-023-06864-2)
Supplement: Supplementary file 1 — Additional file 1: Supplementary table 1. Detailed item and scoring of the QUAC scale. [file 12891_2023_6864_MOESM1_ESM.docx]

Supplementary table 1. Detailed item and scoring of the QUAC scale.

|  | Noh et al. | Spiegl et al. | | McDonald et al. | | Judson et al. | | Lacheta et al. | | Miskovsky et al. | |
| --- | --- | --- | --- | --- | --- | --- | --- | --- | --- | --- | --- |
| 1. Objective stated | 1 | 1 | | 1 | | 1 | | 1 | | 1 | |
| 2. Basic information about sample is included | 1 | 1 | | 1 | | 1 | | 1 | | 1 | |
| 3. Applied methods are described comprehensibly | 1 | 1 | | 1 | | 1 | | 1 | | 1 | |
| 4. Study reports condition of the examined specimens | 1 | 1 | | 0 | | 1 | | 1 | | 1 | |
| 5. Education of dissecting researchers is stated | 0 | 0 | | 0 | | 1 | | 1 | | 1 | |
| 6. Findings are observed by more than one researcher | 0 | 0 | | 0 | | 0 | | 0 | | 0 | |
| 7. Results presented thoroughly and precise | 1 | 1 | | 1 | | 1 | | 1 | | 1 | |
| 8. Statistical methods appropriate | 1 | 1 | | 1 | | 1 | | 1 | | 1 | |
| 9. Details about consistency of findings are given | 1 | 1 | | 1 | | 1 | | 1 | | 1 | |
| 10. Photographs of the observations are included | 1 | 1 | | 1 | | 1 | | 1 | | 1 | |
| 11. Study is discussed within the context of the current evidence | 1 | 1 | | 1 | | 1 | | 1 | | 1 | |
| 12. Clinical implications of the results are discussed | 1 | | 1 | | 1 | | 1 | | 1 | | 1 |
| 13. Limitations of the study are addressed | 1 | | 1 | | 1 | | 1 | | 1 | | 1 |
| Total score | 11 | | 11 | | 10 | | 12 | | 12 | | 12 |

QUACS, Quality Appraisal for Cadaveric Studies)
